# Self-Monitoring Utilization Patterns Among Individuals in an Incentivized Program for Healthy Behaviors

**DOI:** 10.2196/jmir.6371

**Published:** 2016-11-17

**Authors:** Ju Young Kim, Nathan E Wineinger, Michael Taitel, Jennifer M Radin, Osayi Akinbosoye, Jenny Jiang, Nima Nikzad, Gregory Orr, Eric Topol, Steve Steinhubl

**Affiliations:** ^1^ Department of Family Medicine Seoul National University Bundang Hospital Seongnam-si Republic Of Korea; ^2^ Scripps Translational Science Institute La Jolla, CA United States; ^3^ Health Analytics, Research & Reporting Walgreen Company Deerfield, IL United States; ^4^ Department of Digital Health Scripps Translational Science Institute La Jolla, CA United States; ^5^ Digital Health Walgreen Company Bellevue, WA United States

**Keywords:** health behavior, mobile health, mobile apps, reward, self blood pressure monitoring, blood glucose self-monitoring

## Abstract

**Background:**

The advent of digital technology has enabled individuals to track meaningful biometric data about themselves. This novel capability has spurred nontraditional health care organizations to develop systems that aid users in managing their health. One of the most prolific systems is Walgreens Balance Rewards for healthy choices (BRhc) program, an incentivized, Web-based self-monitoring program.

**Objective:**

This study was performed to evaluate health data self-tracking characteristics of individuals enrolled in the Walgreens’ BRhc program, including the impact of manual versus automatic data entries through a supported device or apps.

**Methods:**

We obtained activity tracking data from a total of 455,341 BRhc users during 2014. Upon identifying users with sufficient follow-up data, we explored temporal trends in user participation.

**Results:**

Thirty-four percent of users quit participating after a single entry of an activity. Among users who tracked at least two activities on different dates, the median length of participating was 8 weeks, with an average of 5.8 activities entered per week. Furthermore, users who participated for at least twenty weeks (28.3% of users; 33,078/116,621) consistently entered 8 to 9 activities per week. The majority of users (77%; 243,774/315,744) recorded activities through manual data entry alone. However, individuals who entered activities automatically through supported devices or apps participated roughly four times longer than their manual activity-entering counterparts (average 20 and 5 weeks, respectively; *P*<.001).

**Conclusions:**

This study provides insights into the utilization patterns of individuals participating in an incentivized, Web-based self-monitoring program. Our results suggest automated health tracking could significantly improve long-term health engagement.

## Introduction

The majority of Americans (69%) regularly track at least one indicator of health, including their weight, diet, exercise routine, or symptoms related to chronic disease, with a growing minority (21%) taking advantage of mobile health (mHealth) devices to help them [1]. With an increasing repertoire of mHealth devices, there is a growing trend among many individuals to measure, track, change health behavior, and make health decisions based on quantifiable data collected on oneself. Projections show that the number of everyday wearables, devices, and sensors will increase 5-fold by 2019 [2].

Though the effectiveness of self-monitoring using mHealth technology has been highly variable across studies [3], it is well established that effective self-monitoring can have profound health benefits. For example, among diabetics, blood glucose monitoring is a major component of disease management and provides individuals the ability to assess glycemic targets and evaluate response to therapy [4-6]. Additionally, blood pressure monitoring has been associated with improved short-term blood pressure control and medication adherence [7,8], and self-monitoring has also been shown to improve weight loss and short-term activity levels [9,10]. Importantly, monitoring programs, wearable devices, and other nontraditional health care resources can potentially facilitate healthy behavior changes [11].

As nontraditional health care channels such as retail clinics and virtual care are becoming increasingly popular and beneficial, the traditional health care system is beginning to shift from episode-based fee-for-service to value-based reimbursements [12]. Together, these factors have led to an interest in integrating novel self-monitoring systems into wellness programs, chronic condition management, and the diagnosis of acute episodes. This makes understanding health self-monitoring in these systems an important first step in incorporating these technologies into routine patient care.

In September 2012, Walgreens, one of the largest drugstore chains in the United States, launched its Web-based Balance Rewards for healthy choices (BRhc) program (details in Methods). Members enrolled in the program may track activities and biometric measures to earn points which may be redeemed for purchases at Walgreens. The BRhc Web-based portal and mobile app allows users to set goals and track activities over time. Members can track exercise (including walking, running, and cycling), body weight, and sleep. In April 2014, the program expanded to offer members reward points for connecting biometric devices and inputting blood glucose and blood pressure readings. As a large, nationwide, novel health self-monitoring system, the BRhc offers a unique opportunity to evaluate utilization patterns of individuals enrolled in this incentivized program.

The aim of this study is to evaluate characteristics and activity-tracking patterns for individuals enrolled in the Walgreens BRhc program. Our specific objectives are to (1) present overall participation trends, (2) examine participation across different activities, and (3) explore how automatic activity tracking contributes to utilization patterns.

## Methods

### Program Description

**Figure 1:**
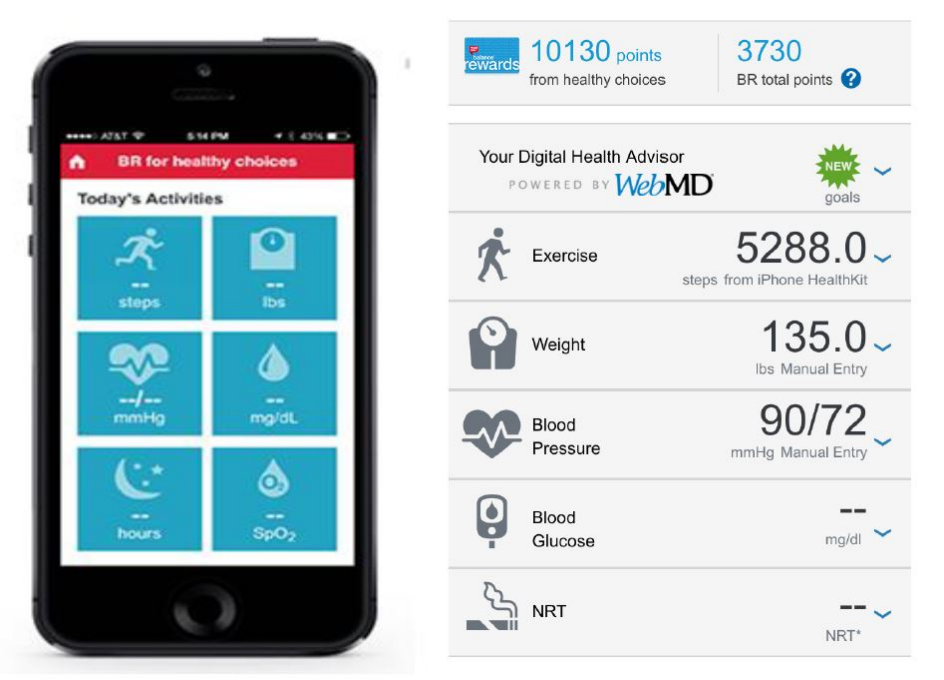
Screenshot of Walgreens Balance Rewards for healthy choices (BRhc) program.

The Walgreens’ BRhc program Web-based user portal can be accessed on its website and via a mobile app as depicted in Figure 1. This program allows members to set goals and track health activities over time. One of the main features of the program is the use of incentives to motivate voluntary participation. Through participation, members receive points that can be redeemed for discounts on purchases.

#### Earning Points in BRhc Program

Members receive points through engaging in a range of activities, including setting initial health goals (250 points), quitting smoking (250 points), filling prescriptions (100 points), and receiving immunizations (100 points). Members also receive points for logging health activities: 20 points per mile walked, ran, or cycled (maximum 1000 points per month); 20 points per day for logging body weight; 20 points per blood glucose test (maximum 40 points per day), and 20 points for logging blood pressure per day.

#### Devices or Apps Linked With BRhc Program

Members have the option of logging these activities manually on the Web-based portal or app, or linking a supported mHealth device or app to their BRhc account for automated data upload (linking a supported technology rewards 250 points). Available apps and devices are presented on Walgreens website, where 22 apps cover fitness trackers, weight loss, medication reminders, blood pressure monitors, blood glucose monitors, or telemedicine, and of the 36 devices, some include fitness and sleep trackers, blood pressure monitors, blood glucose monitors, or pulse oximeter [13].

#### Redemption of Points in Balance Rewards Program

Walgreens Balance Rewards is a loyalty program offered by the Walgreen Company to its customers through earning Balance Rewards points on certain purchases or behaviors through the BRhc program. Integrated Balance Rewards points can be redeemed on most purchases at participating Duane Reade or Walgreens Pharmacy locations. Earned points are converted into redemption dollars at the following tiers: 1000 points = US $1, 2000 points = US $2, 3000 points = US $3, 5000 points = US $5, 10,000 points = US $10, 18,000 points = US $20, 30,000 points = US $35, and 40,000 points = US $ 50. The minimum redeemable is 5000 points for a US $5 reward on a single purchase, and the maximum redeemable per purchase is 40,000 points for US $50. Points expire 3 years after they are earned or if an account has been inactive for 6 months.

### Study Data

Walgreens BRhc utilization data for the entirety of 2014 (January 1 to December 31) was available for this study. This includes data on 7 activity-tracking categories: exercise, weight, sleep, blood pressure, blood glucose, tobacco use, and oxygen saturation. For the purposes of this study, we omitted tobacco use and oxygen saturation as they are less common self-tracking activities [1]. All activity records were either entered manually by users via the Web-based portal, or uploaded automatically using a supported device or app. For each activity recorded, the date and mode of entry (ie, manual or automatic) was available. In total, prior to exclusions, this included 30,420,457 activities recorded from 455,341 unique users. We also collected the age and gender of the users when available.

Exclusion criteria were (1) activities recorded with duplicate values entered on the same day by the same person (2,309,327 activities), (2) users with unknown age or less than 18 years old (n=13,932), (3) users with accounts created before or after 2014 (n=105,849), (4) users who logged their first activity more than 30 days after enrolling in the program (n=3,762), (5) users whose first recorded activity occurred after December 1, 2014 (ie, less than 1 month of follow-up, n=15,700), and (6) tobacco use and oxygen saturation activities (133,101 activities). This resulted in a study population of 315,744 unique users and 12,805,893 activities recorded.

**Figure 2:**
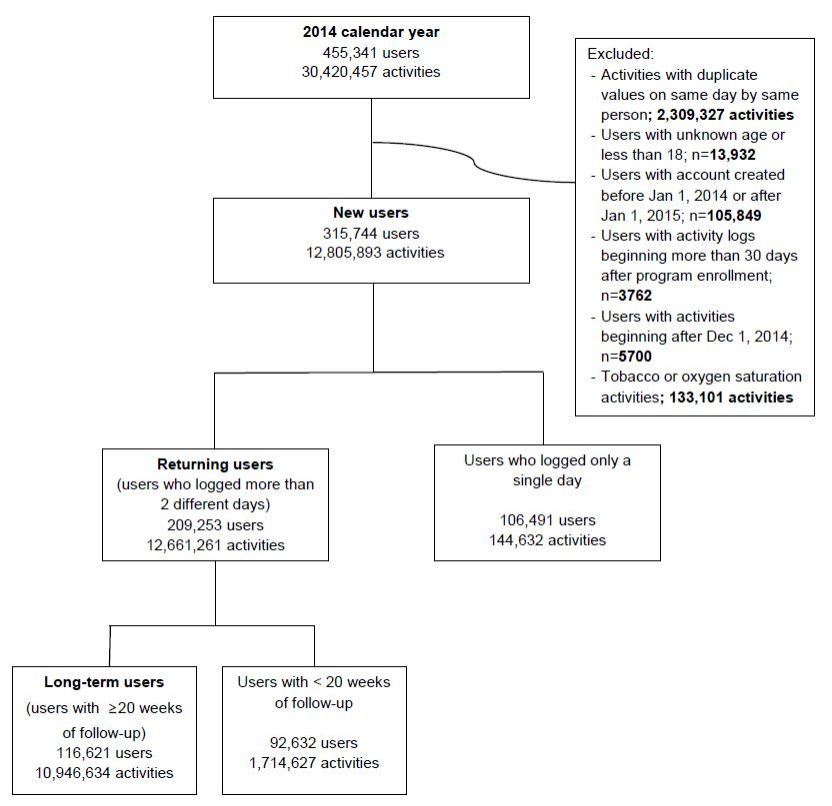
Flowchart of study participants.

We also focused on 2 subsets of users: (1) returning users who recorded an activity on 2 or more different dates (209,253 users, 12,661,261 activities recorded), and (2) users who had at least twenty weeks of potential follow-up (ie, first activity occurred before August 2014; 116,621 users, 10,946,634 activities recorded). A study flowchart is presented in Figure 2. Finally, we differentiated users according to their primary mode of activity entry (ie, manual or automatic).

### Ethical Consideration

Data used in this study comprised gender, age, activity type, date of activity entered either manually or automatically, and amount of activities. These were deidentified datasets and this study was carried out with approval of waiver of informed consent from the Quorum Review Independent Review Board (Review file # 30291/1) for the following reasons: The research involved no more than minimal risk to subjects, the waiver would not adversely affect the rights and welfare of the subjects, and the research could not be carried out without the waiver.

### Utilization Metrics

To assess utilization patterns within the BRhc program, we examined metrics related to the duration users participated in the program and the frequency of activities recorded. We identified the participation length for each user; that is the time between the first and last activity recorded. We also noted gaps between consecutive activities recorded—specifically those exceeding 1 month and 3 months—and defined the length of active participation as the number of weeks users recorded at least one activity. We also computed the frequency of activities recorded for each user over each week of participation, including the type of activity entered (e.g. exercise) and the mode of entry. In addition to presenting the results across and within each type of activity, we also focused on users tracking blood pressure and blood glucose (n=19,143) as they were likely to have hypertension, or diabetes, or be at high risk for these diseases.

### Statistical Analysis

Results are presented as counts, mean (SD), and median (interquartile range) as appropriate. Of the users who recorded an activity on at least two different dates and had at least five months of follow-up, we identified users who recorded activities solely via manual entry and those users who used a supported device or app to upload activities. We compared participation lengths between these groups using *t*-test and Kaplan-Meier analysis. Analyses were performed using SAS version 9.4 (SAS Institute Inc) and figures were created using the *ggplot2* library in R version 2.15.2 [14]. All statistical tests were evaluated at a 2-sided significance level of 0.05.

## Results

### Findings

In 2014, 455,341 unique users participated in the Walgreens’ BRhc program. These users either entered manually or linked a supported device or app that automatically entered 1 of 7 activities tracked in the BRhc program: exercise, weight, sleep, blood pressure, blood glucose data recorded, tobacco use, and oxygen saturation. In total, 30,420,457 activities were entered between January 1 and December 31 (average 66.81 per user). Of the 455,341 users, 315,744 (69.34%) were new users who had at least one month of follow-up activity data. The mean age was 38.65 years (SD 10.95), median was 38.91 (interquartile range [IQR] 31.53 to 42.88) and of the 65.66% (207,330/315,744) of users with nonmissing gender information, 81.71% (169,402/207,330) were women.

Basic demographics and usage characteristics of these 315,744 new members are presented in Table 1.

A large proportion of users (33.73%; 106,491/315,744) who created an account logged in just for a day during the study period. Of the remaining 66.27% of users entering activities for more than 2 days, 77.62% (162,426/209,253) logged activities a month or more after their first entry.

The majority of users (57.00%) of the total study population tracked only 1 type of activity, with exercise being the most common metric tracked (85.52%). However, a sizable number of individuals (21.34%) tracked 3 or more activities – the most common combination being exercise, weight, and sleep. Finally, most users (77.21%) manually logged their activities exclusively through the BRhc Web-based portal, whereas 14.65% of users only logged activities automatically through a supported device or app (8.14% used both means).

### Returning Users

Of the 315,744 new members who enrolled in the BRhc program during the study period, 66.27% (209,253/315,744) of users logged activities on multiple occasions. Usage features of these returning users participating in the program are presented in Table 2.

**Table 1:** Basic usage characteristics among new Balance Rewards for healthy choices (BRhc) members (N=315,744).

Demographic characteristics		n (%)
**Age in years**		
	18-34	105,070 (33.28)
	35-49	164,345 (52.05)
	50-64	39,073 (12.37)
	≥65	7256 (2.30)
**Gender**		
	Female	169,402 (53.65)
	Male	37,928 (12.01)
	Unidentified	108,414 (34.34)
**Participation length**		
	1 day	106,491 (33.73)
	<4 weeks	46,467 (14.72)
	4≤weeks<20	115,598 (36.73)
	≥20	46,828 (14.83)
**Activity logged**		
	Exercise	270,036 (85.52)
	Weight	129,566 (41.03)
	Sleep	105,582 (33.44)
	Blood pressure	34,013 (10.77)
	Blood glucose	18,705 (5.92)
**Number of activities**		
	1	179,988 (57.00)
	2	68,371 (21.65)
	≥3	67,385 (21.34)
**Source of logging**		
	Web-based portal	243,774 (77.21)
	Device or app	46,262 (14.65)
	Both	25,708 (8.14)

**Table 2:** Weekly usage characteristics among returning users across activities logged.

Demographic characteristics	Any	Exercise	Sleep	Weight	Blood pressure	Blood glucose
N	209,253	186,037	62,040	56,533	16,318	9737
Age in years, mean (SD)	38.84 (10.84)	38.98 (10.61)	38.97 (12.35)	38.36 (12.48)	41.26 (13.63)	40.66 (13.71)
Female, n (%)	109,826 (52.48)	91,565 (49.22)	46,493 (74.94)	43,311 (76.61)	12,182 (74.65)	7093 (72.85)
Male, n (%)	26,047 (12.45)	22,972 (12. 35)	10,869 (17.52)	9291 (16.43)	2950 (18.08)	1915 (19.67)
Participation length, median (IQR)	8 (4-18)	8 (4-18)	10 (3-21)	7 (2-16)	6 (2-15)	6 (2-14)
Activities logged/week, mean (SD)	5.66 (7.73)	3.62 (4.90)	4.44 (4.73)	2.84 (3.44)	3.53 (4.23)	5.57 (6.27)
Days of activity logged/week, mean (SD)	2.24 (2.12)	2.27 (2.19)	1.87 (1.63)	1.48 (1.40)	1.79 (1.59)	2.19 (1.74)
Gap > 1 month (%)	49,641 (23.72)	45,232 (24.31)	18,502 (29.82)	15,063 (26.64)	4063 (24.90)	2126 (21.83)
Gap > 3 months (%)	11,385 (5.44)	9159 (4.92)	5902 (9.51)	6060 (10.72)	1332 (8.16)	661 (6.8)
Device or app (%)	69,506 (33.22)	66,489 (35.74)	32,424 (52.26)	9020 (15.96)	414 (2.5)	127 (1.3)

The median length of participation for these users was 8 weeks (IQR 4-18). There was a tendency among users who logged exercise and sleep activities to participate in the program the longest (median 8 and 10 weeks, respectively), whereas users who logged blood pressure and blood glucose had the shortest participation duration (median 6 weeks; all *P*<.001). A proportion (23.72%) of users had a moderate (at least one month) gap between consecutive logged entries over their participation period, and a small number of users (5.44%) had a substantial gap (at least three months) between entries. Overall, half of the returning users participated for at least eight weeks, but periods of inactivity were not uncommon.

Meanwhile, returning users logged 5.66 activities per week (SD 7.73) when all different types of activities were included, but median value of logged activities was 2.87 (IQR 0.52-7.67), indicating a small percentage of highly-active users contributed to the increase in the mean frequency. Among specific activities, blood glucose had the highest weekly entry (mean 5.57 entries per week) and body weight had the least (mean 2.84 entries).

Finally, we note that a higher proportion of these returning users used a supported device or app compared to all new users (33.22% vs 22.79%, respectively). The most commonly used device was Fitbit (59.89% of returning users with supported device or app; 41,608/69,472), followed by Jawbone (2.39%; 1666/69,472) and Misfit (1.60%; 1112/69,472), which are activity trackers, wireless-enabled wearable technology devices that measure data such as steps walked, heart rate, or sleep time. The commonly used apps were Runkeeper (20.03%; 13,917/69,472), Lose It! (10.75%; 7472/69,472), MyFitnessPal (7.29%; 5062/69,472) and MapMyFitness (6.41%; 4456/69,472), which also tracked caloric intake, calories burned, and weight. Furthermore, the vast majority (96.57%; 69,506/71,970) of new users who linked a device or app returned to logged activities on subsequent days. However, in part due to the availability of supported tools only for specific activities, we observed vast variability across activities. There is potential that this variability in linked device or app use accounts for the utilization differences between activities observed above. We further examine this hypothesis in more detail in the following sections.

### Long-Term Utilization

**Figure 3:**
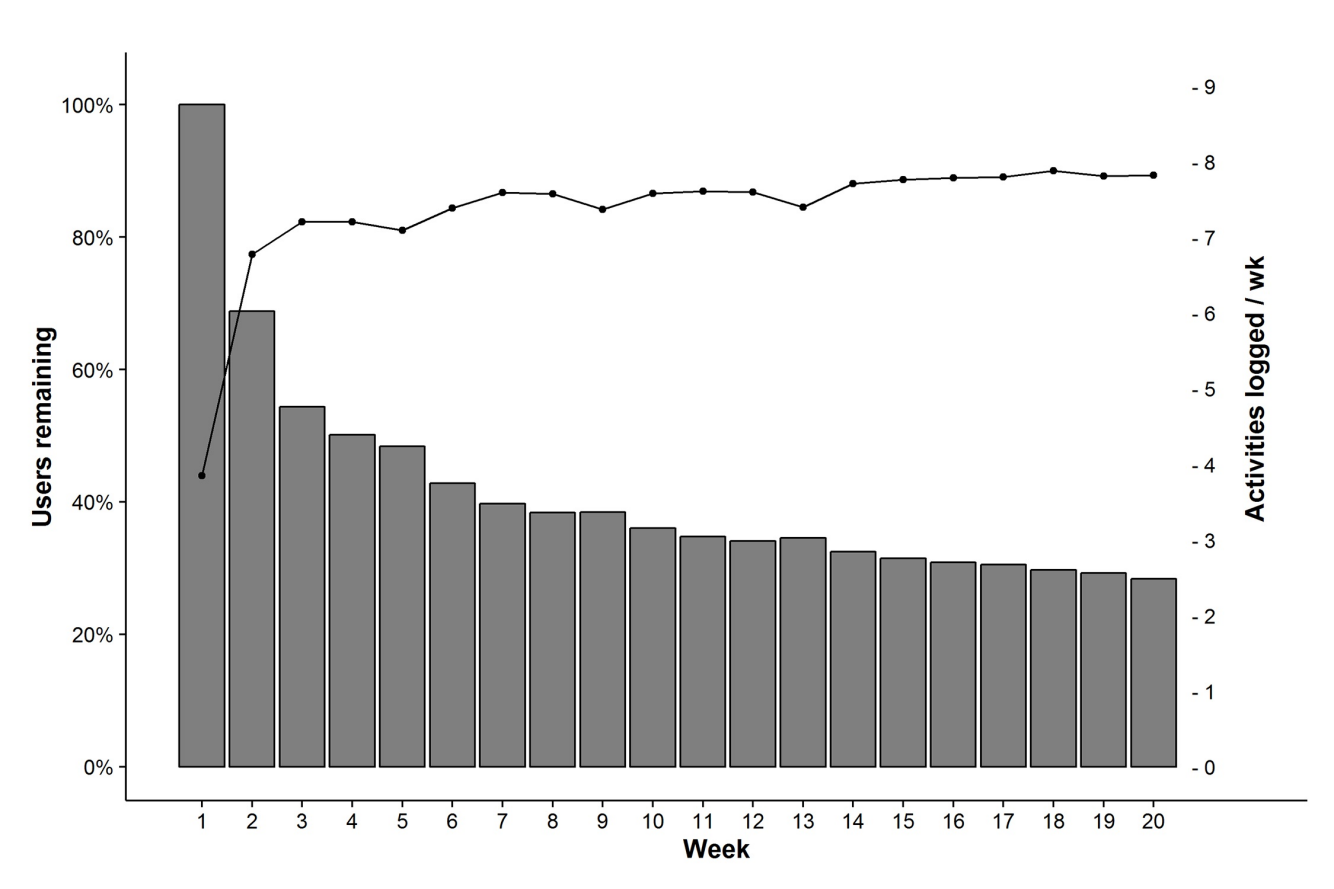
Percentage of users still logging activities after certain number of weeks, and the average number of activities logged during that week. Bar: users remaining, line: activities logged per week.

To explore long-term usage, we identified a subset of members (116,621 users with 10,946,634 recorded activities) who joined the program before August 2014 and who had 2 or more log-on dates. This allowed us to examine utilization over the first 20 weeks after program enrollment. Of these users, 31.20% (36,390/116,621) stopped participating after 1 week, and 49.88% (58,177/116,621) stopped within 1 month. However, after this initial dropout, the number of users in the program remains fairly consistent over many weeks (Figure 3).

After 20 weeks, 28.36% (33,078/116,621) of registered users were still actively engaged in the program. Meanwhile, combined with the duration of program participation, the frequency of program participation over the first 20 weeks demonstrated some interesting trends. First, the average number of activities logged by users was 4.28 during the first week in the program. However, after excluding the roughly one-third of users who ceased recording activities after 1 week, the average number of activities logged by participating users increased to 7.53 by the second week. After 4 weeks, this number was 8.01 and remained relatively steady throughout the 20-week period examined (Figure 3).

Overall, this demonstrates that while a large proportion of users stopped participating in the BRhc program early on (roughly half by 4 weeks), those that did continue to log activities did so at a fairly consistent level throughout their participation period. We observed that users log activities roughly three days a week, on average, the most common activity being exercise.

### The Role of Supported Devices and Apps That Automatically Log Activities

Around one-third of returning users and 23% of all users used a supported device or app which, when linked to the account, was able to record and automatically upload activities directly to the BRhc portal. There was also marked variability in the proportion of users who used such tools across different activities. For example, 35.74% of users (66,489/186,037) logging exercise activities used an automatically uploading device or app while 52.26% (32,424/62,040) of users logging sleep did too.

**Figure 4:**
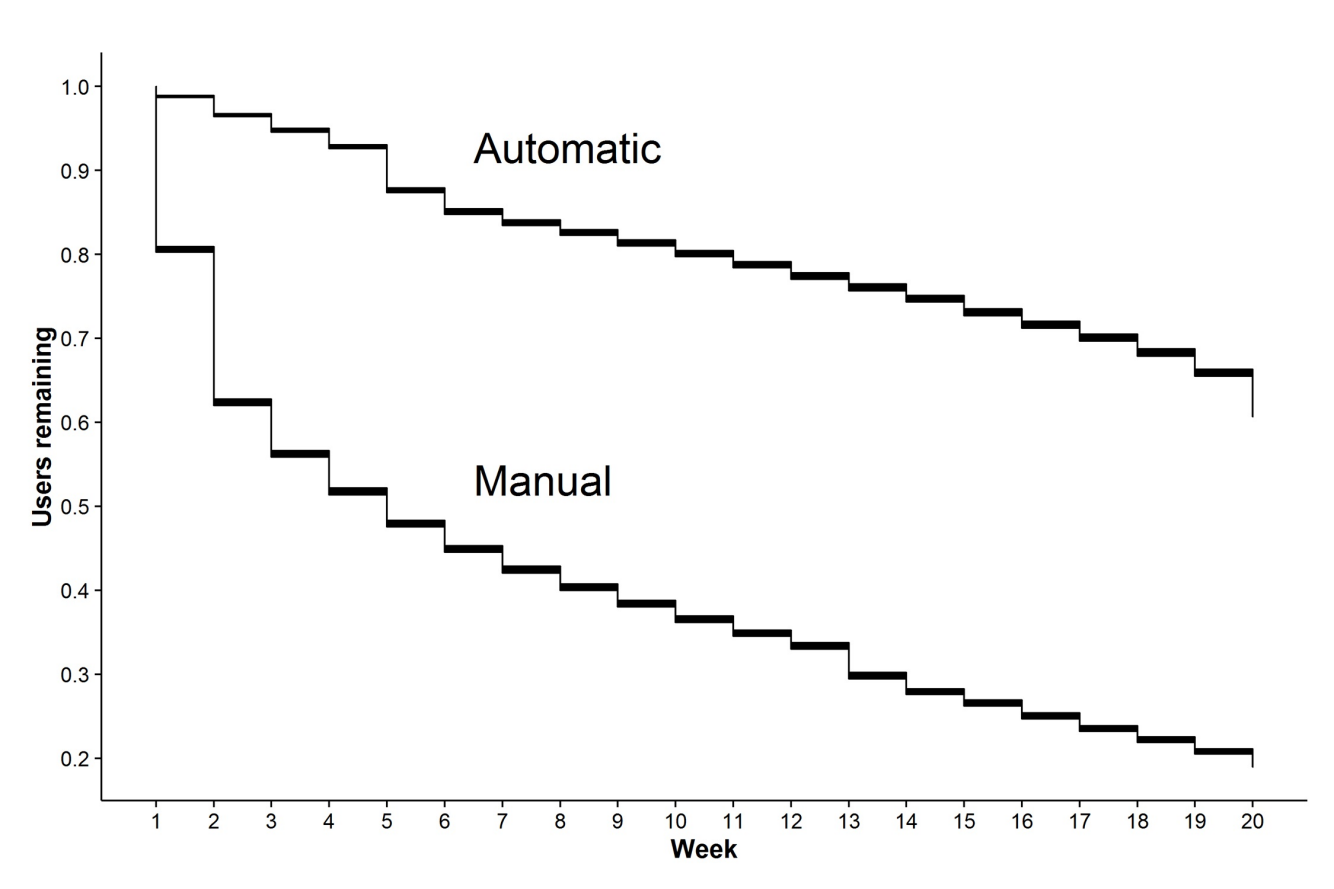
Kaplan Meier survival curve with 95% confidence intervals for the duration of participation among users logging activities manually through the online portal or automatically using a linked device or app.

Among users who joined the program before August 2014, automatic activity logging was strongly associated with longer participation length (Figure 4).

Users logging activities automatically using a linked device or app participated on average (mean) 24.01 weeks versus 10.54 weeks among those logging activities manually (*P*<.001). Furthermore, users automatically logging activities were active participants (number of weeks recording at least one activity) for 20.15 weeks on average compared with 5.23 weeks (*P*<.001). This trend was consistent across all the tracking activities where automatic upload was common: exercise (20.41 weeks vs 5.71), sleep (13.61 vs 3.25), and weight (10.76 vs 5.18; all *P*<.001) but not for blood pressure (6.26 vs 6.18; *P*=.88) or blood glucose (8.37 vs 6.71; *P*=.11) where automatic tracking was rare (Table 3).

Although most of the users were female, male participants were more likely to be the active users in this program, especially in weight tracking (mean active weeks of male vs female in manual, 6.09 vs 5.02; *P*<.001, values in automatic upload, 14.44 vs 9.52; *P*<.001).

**Table 3:** Mean (SD) of participation length (weeks) between users logging activities using the Web-based portal (manual) or through a supported device or app (automatic).

Type of activity		Manual, mean (SD)			Automatic, mean (SD)			*P* value
		Total	Female	Male	Total	Female	Male	
Any	Participation	10.54 (11.64)	10.28 (11.67)	11.72 (12.15)^a^	24.01 (13.47)	24.81 (13.14)	25.30 (13.74)^a^	<.001
	Active participation	5.23 (6.78)	5.14 (6.67)	5.91 (7.73)^a^	20.15 (13.18)	20.71 (12.96)	21.80 (13.61)^a^	<.001
Exercise	Participation	11.34 (11.80)	11.06 (11.90)	12.62 (12.16)^a^	24.03 (13.64)	24.89 (13.22)	25.20 (13.75)	<.001
	Active participation	5.71 (6.96)	5.67 (6.93)	6.22 (7.59)^a^	20.41 (13.22)	21.07 (13.00)	21.85 (13.60)^a^	<.001
Sleep	Participation	8.65 (9.00)	8.67 (8.96)	8.68 (9.40)	21.69 (10.98)	21.58 (10.87)	22.21 (11.35)	<.001
	Active participation	3.25 (3.28)	3.20 (3.14)	3.57 (4.09)^a^	13.61 (10.08)	13.43 (9.97)	14.21 (10.48)^a^	<.001
Weight	Participation	11.66 (11.54)	11.63 (11.43)	11.96 (12.16)	16.41 (11.99)	15.23 (11.31)	20.04 (13.32)^a^	<.001
	Active participation	5.18 (6.57)	5.02 (6.24)	6.09 (8.16)^a^	10.76 (9.53)	9.52 (8.48)	14.44 (11.41)^a^	<.001
Blood pressure	Participation	11.81 (10.61)	11.64 (10.44)	12.68 (11.31)^a^	10.76 (8.96)	10.39 (7.89)	11.19 (9.89)	.19
	Active participation	6.18 (7.39)	5.87 (6.95)	7.59 (8.98)^a^	6.26 (5.67)	6.04 (5.13)	6.46 (6.13)	.88
Blood glucose	Participation	11.58 (10.68)	11.31 (10.49)	12.66 (11.30)	11.74 (8.25)	9.94 (6.91)	14.00 (9.33)	.91
	Active participation	6.71 (8.00)	6.28 (7.54)	8.33 (9.37)^a^	8.37 (7.02)	7.62 (6.44)	9.33 (7.71)	.11

^a^Variable with significant difference between female and male.

Although the majority of all users (77.21%; 243,774/315,744) exclusively logged activities manually through the BRhc Web-based portal, interestingly, automatically-entered data accounted for the majority of all recorded activities. Of the nearly 13 million total activities recorded by returning users, only 23.46% (2,969,761/12,661,261) were manually entered, while the remaining 76.54% (9,691,500/12,661,261) were entered automatically using a linked device or app. Again, we observed that sleep (92.02%; 2,430,157/2,640,769) and exercise (83.00%; 6,966,901/8,393,429) had the highest frequency of activities logged automatically while weight, blood pressure, and blood glucose were lowest (all *P*<.001) among users enrolled prior to August 2014.

### Users Tracking Blood Pressure and Blood Glucose

BRhc members logging blood pressure and blood glucose recordings may represent a slightly different population than the other BRhc members as they would be more likely to have hypertension, diabetes, be at increased risk, or have the perception to be at risk for these conditions. Furthermore, automated data upload to the BRhc platform using a device or app was rarely used for tracking blood pressure or blood glucose in 2014. However, these users could benefit from the program, as monitoring of blood pressure and blood glucose is critical to the control of hypertension and diabetes.

A total of 19,143 returning users in the BRhc logged blood pressure or blood glucose measurements. Similar to the entire BRhc member population, their average age was 41.62 (SD 13.61) years and 80.25% (14,290/17,806) were female. On average, users logging blood pressure or blood glucose activities tracked a wider range of activities than other users (median 4 vs 2). Most of these users also tracked weight (92.73%; 17,751/19,143), exercise (91.36%; 17,489/19,143), or sleep (86.34%; 16,529/19,143). There were 12,401 new users tracking blood pressure or blood glucose that enrolled in the BRhc program prior to August 2014. Similar to the entire population, 27.02% (3351/12,401) of these users quit logging activities by the first week and 21.84% (2709/12,401) were still recording activities after 20 weeks.

## Discussion

### Principal Findings

Monitoring of physiologic parameters, health activities, and health behaviors outside of the medical setting has the potential to enable alternative systems of health management that can be both more individualized and convenient for health consumers. An understanding of the patterns of home-based self-tracking can provide insights into optimizing such programs in future health care models.

The Walgreens’ BRhc program is one such alternative health management system that, in 2014, enrolled 455,341 members. Walgreens incentivizes users to log health-related activities that can be tracked using the BRhc portal. Recently, a study was performed to increase physical activities using wearable devices and gamification from incentivized consumers [15]. This study analyzed the descriptive data from GOODcoins, a self-guided, consumer engagement and rewards platform incentivizing physical activities. The results suggested that challenges and incentives might work for connected and active participants in achieving healthy physical activities. Our study showed consistent, extended results to the previous finding of how incentivized consumers track health behaviors and health data in real-world setting with large population.

In this study we examined the utilization characteristics of these individuals.

First, BRhc users who provided their gender information were mostly women (81.7%; 169,402/207,330) in their thirties (median age 38.9 with IQR 31.5 to 42.9). This finding is consistent with previous studies of Web-based weight loss programs [16,17]. A recent survey from the National Cancer Institute found that being younger and female were associated with increased use of Web-based and other nontraditional health resources [18]. Considering the size of participants and impact of availability of Walgreens drug stores, this phenomenon could be representative of real-world setting, not artificially chosen nor controlled population.

We note that BRhc members were self-selected without any mediation of, for example, health care professionals. Thus, strategies to better engage men [19] and the elderly [20] need to be more fully explored in order for home-based health management to become a more common occurrence in everyday lives across all demographics. Also, in our analysis of a long-term observation group showed that men, although being a small portion of the total users, who voluntarily selected the program tended to be more active users in tracking health behaviors than women. This trend seemed prominent in weight tracking where intentional efforts are needed more than any other activity, even when linked with apps or devices. So engagement methods need to be studied differently according to gender.

We presented frequency and duration utilization metrics of the BRhc program. Approximately one-third of users created an account, logged a single activity, and then never returned. These users reflect an audience with initial interest, but are either immediately turned off or fail to see (or care for) any benefit of continuing to log activities in the program. This result follows a trend seen in other studies, for example: one study of a weight loss app found that it was downloaded by nearly 190,000 users but only 2.6% used the app for at least one week [21]; and another showed high attrition rate and rapid decrease in usage in mobile and Internet programs for maintaining physical activity after cardiac rehabilitation [22]. Especially in the era of consumer-centric mobile or Web-based intervention studies, phenomenon of dropout attrition and nonusage attrition where substantial proportion of users not using the intervention as guided or dropping out before completion, the so called “law of attrition” [23] still remains the challenging issue. Our attrition curve also suggested early “curiosity plateau,” followed by a more steady group of users who remained in the program. In this regard, we believe that identification and understanding of the characteristics of these individuals, and factors that promote interest or motivate behavior change is critically important in this rapidly evolving field.

### Factors Associated With Adherence in Healthy Behavior Program

According to a review regarding Web-based recruitment methods for mobile health study, virtual aspect of intervention might lead to comfort in enrolling the trial, less investment in ongoing usage, and possibility of fraudulent enrolling in the trial [24]. Other proposed factors that can influence patient retention and engagements are usability of the program, interactive feedback, tangible and intangible observable advantage in using the program, effort and time required, networking effects or peer pressure, and user factors (demographic education, previous experiences) [23]. To increase a consumer’s motivation and active participation, various incentive-driven mobile health technologies such as education, reminder, feedback, social, financial, or gamification can be simultaneously used and provide its efficacy [25].

In order to test or implement a mobile health program with actively-engaged users, attrition must be actively reported in metrics such as usage half-life, dropout attrition curve, or Kaplan-Meier analysis [23] and analyzed according to user factors (sociodemographic, health condition etc), usability, and components of the program itself for solving the attrition problem.

Also for the behavior change, it is not clear whether a previous active tracker is viewed as a superuser in the BRhc program or a person who does not participate in health behavior tracking has become an active tracker initiated by the program. Further exploration around behavior changes is needed to clarify the impact of behavior changes on users at different stages.

Michie et al developed and refined the behavioral change technique taxonomy for behavioral change intervention [26], and this system can be effectively adopted for implementing and evaluating a mobile health intervention program. In the BRhc program, incentives such as rewards points, goal setting, and self-monitoring of behavior were used as behavior change techniques motivated by Fogg’s behavioral change methods [27,28]. This program definitely proved its positive impact on pervasiveness recognized by 800,000 users, 250,000 connected devices, and 73 miles logged as of April 2015 [29], but further issues with continuous engagement or participation of various ages with gender seemed to be solved.

### Long-Term Adherence to Healthy Behavior Program and Automated Self-Monitoring Tool

Our results of long-term utilization demonstrated that roughly one-third of returning users quit within 1 week, half quit within a month, and two-thirds quit by 5 months. However, the attrition rate declined rapidly after this, particularly so in users logging activities using a linked device or app. Over half (57%) of all users that were still participating after 1 month continued to participate for at least twenty weeks. Since motivating behavior change to improve health management requires continuous and often complex processes, engaging consumers both initially and for prolonged lengths of time will be important components of success. There is still much to learn about motivating long-term participation, but at a minimum, tools should be simple enough for users and incorporate proven behavior change theories through the use of rewards or incentives [30].

One such tool to improve long-term health self-monitoring is mobile and wireless health-tracking technologies. These technologies can collect, transmit, and aggregate health data – automated, thus removing this burden from the user. We discovered that users tracking data using devices participated in the program, on average, 24 weeks compared with 11 weeks among users not using any device. Furthermore, these users were active 20 weeks on average compared with 5 weeks. Another study looking for adherence to the protocol through mobile phone apps which compared website or paper diaries for weight loss also proved the advantage of mobile phone apps even when it was not a fully automated process [31].

More advanced and user-friendly self-monitoring tools are continually being developed, and their capacity to interact with and be interpreted within traditional (eg, electronic health records) and nontraditional health care systems will be critical in their implementation. It is becoming a common feature of many new mobile health devices to enable automated collection, downloading, and sharing of measured biometrics. Yet, while our study showcased the benefit of automated systems, we feel the next frontier in this field needs to address the interpretation of data collected from these devices beyond displaying the data to users in attractive pictures [32]. Many studies have shown the benefit of remote monitoring in improving outcomes with patients not only in chronic condition such as chronic obstructive pulmonary disease, heart failure, diabetes, or hypertension [33,34] but also meaningful interpretation of data at the point of need can be valuable in acute infectious disease such as Ebola outbreak [35].

### Individuals With Chronic Condition and Automated Self-Monitoring Tool

Individuals with chronic conditions could potentially benefit most from automated tracking tools. Health complications from hypertension and diabetes, for example, are largely preventable with proper management. However, these conditions (and others) are often poorly controlled. In a number of cases, automated interventions have shown health benefits: a prior study showed that a fully automated behavioral intervention leveraging Web, mobile, and automated mobile phone calls significantly improved glycemic control, body weight, and diabetes risk among prediabetics [36]; another showed that a physical activity intervention consisting of automated weekly exercise scheduling reminders, a message board to share their experience with others, and feedback on their level of physical activity increased and maintained levels of physical activity in healthy adults [37]; and another showed that a fully automated smoking cessation program using email, Web, interactive voice response, and short messaging service was associated with abstinence rates without the use of nicotine replacement therapy [38]. However, even with access to the latest technologies to monitor any biometric or condition, engagement, which leads to behavior change, is key. Technology in and of itself is unlikely to drive change toward positive health outcomes. Additional factors, like incentives used in the BRhc program and how they interact with technologies to engage participants have shown positive health behavior changes [39] and potential to drive the future of health self-monitoring.

### Limitations

This study focused on characteristics of users of an incentivized, Web-based self-monitoring program. Although users of the BRhc program can be representative of real-world setting, when we look at long-term utilization, the composition of users—self-selected, young, and most likely female—as well as high attrition rate affect the validity and generalizability of our findings. Further exploration of the relationship between utilization patterns and their impact on perceived value, especially among users tracking blood pressure or blood glucose, are needed to better understand the potential impact on behavior change and chronic conditions management.

Since this program used incentive for behavior change and engagement, relationship between Balance Reward points and usage activities should have been investigated. Balance Rewards points are calculated both from purchasing certain products and earning behavioral points from the BRhc program to be used as redemption. However, since the variables of total reward points or redemption contents were not available in our database, and hypothetical BRhc program can be calculated from usage activities itself, it was difficult to prove the role of “incentive” leading to behavior changes. Also incentive itself was relatively small. If a first-time user creates an account, sets up a goal, and logs in 9 different activities for 1 month, not skipping even one, he or she will get maximum 6050 points, which can be redeemed at 6 dollars a month.

### Conclusions

Web-based and mobile health self-monitoring is popular in the general population, and could play a critical role in the future of health management and wellness. Self-monitoring has been shown to improve health and management of chronic conditions. However, there are considerable challenges in initiating and sustaining engagement for long periods of time. This study provides insights into utilization patterns of incentivized users participating in a large, nationwide, Web-based self-monitoring program and supports the benefit of automated health tracking to help maintain long-term engagement.

## Abbreviations

BRhc: Balance Rewards for healthy choices
mHealth: mobile health
